# Online application for self-referral of the patients with breast symptoms

**DOI:** 10.1016/j.amsu.2021.102372

**Published:** 2021-05-08

**Authors:** Ahsan Rao, Humayun Razzaq, Ben Panamarenko, Alex Bottle, Azeem Majeed, Emma Gray

**Affiliations:** a Department of Surgery, Southend University Hospital NHFT, Southend on Sea Essex, UK; b School of Public Health and Faculty of Medicine, Imperial College London, UK; c Department of Public Health and Primary Care, Imperial College London, UK; d Department of Breast Surgery, Southend University Hospital NHFT, Southend on Sea Essex, UK

**Keywords:** Self-referral breast application, Breast complaints in GP, Breast care awareness, Urgent breast clinic appointment, Breast cancer pathway

## Abstract

**Introduction:**

The study aimed to devise a self-referral mobile/web application for patients with new breast symptoms, giving them an outcome, thus bypassing the need for primary care consultation.

**Methods:**

The online application was designed on the automated algorithm based on evidence-based guidelines for referral to breast onco-plastic units. A retrospective questionnaire-based anonymous survey was carried out at the breast unit in Southend University Hospital (January 2019 to March 2020). The outcome of the patients was recorded, the same data was entered in the software and its outcome was compared with their clinic outcome to assess and validate the software. Chi-square and *t*-test were used in formulating results.

**Results:**

Data was collected for 366 patients who were referred urgently to the clinic. Only 50.5% (n = 186) were appropriately referred, with the main complaint being breast lump (94.1%). 39.6% of referred patients did not require a secondary care referral. Sensitivity and specificity for identifying patients requiring urgent referral was 100% and 98%, respectively.

**Conclusion:**

A significant number of urgent referrals to breast units do not require urgent specialist referral, and this results in a big strain on the hospital service. The discussed self-referral pathway is a promising alternative with the potential to reduce workload in primary and secondary care and improve patient satisfaction.

## Highlights

• Half of urgent GP referrals to Breast oncoplastic units were inappropriate.
• The proposed self-referral mobile application had 100% sensitivity and 98% specificity.
• The app will allow to choose and book appointment thus improving compliance.
• The app will reduce inappropriate referrals and will cater to patient satisfaction .

## 1 Introduction

Breast cancer remains the most common cancer in females in the United Kingdom, with over 36,000 new cases annually [1]. These patients will frequently present to primary care, with one UK study reporting that a general practitioner (GP) will see an average of two breast complaints every fortnight [3]. Rates of referrals from GPs remain high despite the introduction of the “two week wait” initiative in 1999, with the aim to aid GPs to stratify urgent and non-urgent referrals to ensure patients with suspected cancer symptoms are seen within two weeks. One study concluded that one third of referrals to ‘one stop breast clinics’ were inappropriate and therefore put unnecessary strain on the service [4].

To enable suspected breast cancer patients to be seen in the quickest possible time and avoid overwhelming urgent services further initiatives must be taken. Self-referral to specialist services allows patients with symptoms as defined by guidelines to bypass the stage of needing a referral from a GP to a specialist, consequently allowing patients with red flag symptoms to be seen first. It is practised among other specialties already [5]. Self-referral to physiotherapy services is increasingly common, with high satisfaction in the service reported by the specialty and GPs, with associated financial benefit [5,6]. The workload of GPs has increased significantly in recent times, mainly due to an ageing population and people more commonly having multiple co-morbidities requiring ongoing care. The increased waiting time for appointments with GPs has been the subject of much discussion recently, and self-referral pathways aim to alleviate some of this burden.

The current COVID-19 pandemic has drastically affected the working of primary care services. A large number of face-to-face consultations are suspended. The waiting time to see a GP is prolonged even more for patients with breast symptoms. In view of the success of existing systems for other services, we have developed an online self-referral portal based on current NICE guidelines for breast disease [7]. The patients can use this portal on their smart phones or computer to refer themselves directly to the specialist breast unit. Based on the medical history provided by the patient, the online portal can then categorize patients into those who need a two-week wait urgent appointment, those who need a routine appointment and those who can be managed by the GP. The aim of the study was to develop and validate the online portal for the self-referral of the patients with new breast symptoms.

## 2 Methods

The initial phase of the study was to conduct a retrospective questionnaire-based survey on the patients who attended urgent symptomatic breast clinics. The adult patients over the age of 16 who attended urgent symptomatic breast clinics in the breast unit at Southend University Hospital between January 1, 2019 and March 1, 2020 were contacted by telephone. They were either asked to complete an online questionnaire or the online questionnaire was completed on their behalf while on the phone by the researcher who called them. The details of the study were provided, and informed consent was obtained over the phone. The questionnaire was developed by the researchers of this study.

The online questionnaire consisted of 34 questions *(*Appendix 1*).* Clinic notes and previous consultation letters were referred to where required in order to obtain accurate and specific information. They ranged from obtaining information about nature of presenting complaint to duration of onset, associated symptoms and signs, any red flags, gravida status including number of children, age at giving first birth, any miscarriages or termination of pregnancies as well as history of breast feeding. It also included questions about family or personal history of breast or ovarian cancer as well as any other cancer syndromes, age of menopause, use of hormonal contraceptives, psychiatric history, medical background including drug history, any self-examination findings and any history of obesity.

The questions in the questionnaires were designed by the researchers of this study to obtain the information necessary to meet referral guidelines to urgent symptomatic clinics [14]. It also included questions that may improve the ability to predict those patients in the clinic who have a suspicious lump or other symptoms that warrant biopsy. It is important to obtain this information as a patient with suspicion of cancer on physical examination or imaging both will indicate the true need to be seen in the urgent breast clinic. Once the data from the questionnaire was obtained, further information from electronic medical records was gathered to assess the patients who had a biopsy in the clinic and what was the result of the biopsy. This way, the study population was divided into two groups: those who had a biopsy in the clinic with suspicion of cancer indicating their importance of being seen urgently by the breast team and those patients without a biopsy taken in the clinic and discharged. The response to each question was then compared between the two groups. This information was obtained to devise the software for self-referral, as any question that would suggest a significant difference in the two groups could be used in the designing the question algorithm in the software.

The online software was designed based on the information provided by the national referral guidelines and other relevant information obtained from the questions in the questionnaire that were linked to the patients who had a biopsy in the clinic *(*Appendix 2*).* The software (www.eastenglandbreastcare.uk) asks six questions from the patient and uses it to provide an outcome of whether the patient should be seen urgently in the breast clinic. The software generates four outcomes for referral: to be seen urgently within two weeks in the symptomatic breast clinic, to be seen routinely in the symptomatic breast clinic, to be seen in the family history clinic and to be managed by the GP in the community.

Once the software was created, the information of the study participants was added individually and it generated outcomes for each patient. There were four choices of outcomes: to be seen urgently within two weeks in the symptomatic breast clinic, to be seen routinely in the symptomatic breast clinic, to be seen in the family history clinic and to be managed by the GP in the community. The breast surgeons were blinded by the outcomes provided by the software. The outcomes of the software for each patient was validated by comparing it with the outcomes provided by the breast surgeons.

Descriptive statistics were conducted on the study population for their responses to the questionnaire. The categorical and continuous variables were compared between the groups using chi-square test and *t*-test, respectively. The breast surgeons went through all the information from the patients provided in the questionnaire and selected those patients who required an urgent 2 week wait review by the breast team. This acted as a standard to assess the accuracy of the software in evaluation of the patients who were provided urgent outcome review. It was important to check that the software was accurate in assessing those patients who require urgent clinic review because these are the one with high risk of developing cancer. The study satisfies the criteria for SQUIRE 2.0 (Standards for Quality Improvement Reporting Excellence) [14].

## 3 Results

There were a total of 366 participants who responded to the questionnaire and were included in the study. The response rate for the questionnaire was 50.8%. The average age of the patients was 47.6 (SD 16.1) and majority of them were females (n = 340, 92.8%). The most common presentation was breast lump (n = 213, 58.2%), breast pain (n = 71, 19.4%), nipple pain (n = 23, 6.3%), skin changes on breast (n = 20, 5.5%), nipple discharge (n = 10, 2.7%), lump in the armpit (n = 6, 1.6%), and nipple retraction (n = 5, 1.4%)*.* The average age of having the first child was 26.0 (SD 7.3). Most of the women who had had their menopause had it before the age of 50 (n = 82, 65.1%). The mean time in months for the duration of breastfeeding was 8.2 (SD 12.0). There were 8.8% (n = 32) and 3.0% (n = 11) of the patients had first degree relatives with a family history of breast and ovarian cancer, respectively. Twenty percent (n = 20) and 11.5% (n = 43) of the patients had previous diagnosis of breast cancer and non-cancer breast conditions. Few patients had previous breast surgery (13.5%, n = 51) and the common operations were breast implant (n = 20, 38.5%), excision of benign lump (n = 13, 25%), wide local excision (n = 10, 13.2%) and mastectomy (n = 5, 9.6%). The average time for patients taking either oral contraception or hormone replacement therapy was 5 years. A small proportion of patients had diabetes (n = 24, 6.5%), were taking immunosuppressants including steroids (n = 19, 5.1%), or obese (n = 68, 18.1%).

The patients who had a biopsy (n = 24, 6.6%) for suspicious breast lesions on examination or imaging, 22 (90.1%) of them were found to have breast cancer, and 2 (9.9%) of them were found to have benign breast changes. Their main complaints of the patients who had suspicion of cancer and had biopsy in the clinic were mainly breast lump (n = 22, 90.9%) or nipple changes (n = 2, 9.1%). Compared with those patients who did not have any biopsy in the symptomatic clinic, the patients who had the biopsy had a significantly higher average age (59.3 vs. 46.6, P < 0.001). There was no significant difference in any other variable assessed in the questionnaire between the two groups, patients with suspected cancer and biopsy and those who did not have biopsy.

**Fig. 1:**
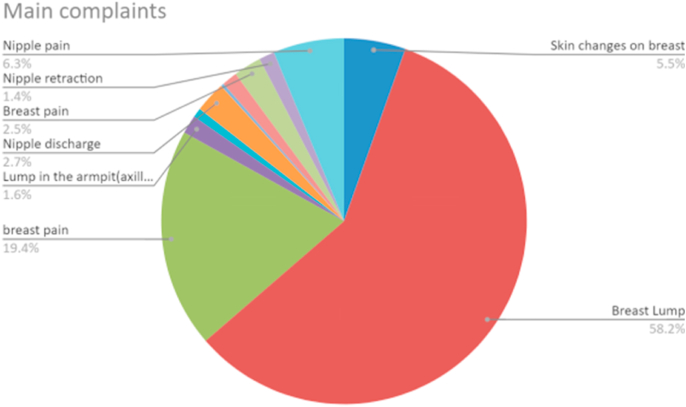
The common complaints by the patients presenting to the symptomatic breast clinic.

All referrals made by the GP were urgent, that is, to be seen within two weeks, on the two-week wait cancer referral pathway. Only 50.8% (n = 186) were appropriate according to the guidelines for referral pathway. Their main complaint was breast lump (n = 175, 94.1%) and 5.9% (n = 11) had nipple changes (nipple retraction/distortion/rash/discharge). Of the patients (n = 180) that did not require an urgent referral, 35 (19.4%) of them should have been seen in routine non-urgent breast clinics and 145 patients (80.5%) should not have been referred to secondary care and should have been dealt in the community. Of the patients who did not require secondary care referral (n = 145), 64.9% presented with breast pain, and other common presentations were breast lump (24.8%) and general concern about breast cancer (6.2%) (see Table 1, Fig. 1).

**Table 1:** Comparison of prevalence of variables in two groups (patients who had biopsy for suspicion of breast cancer and those who were not suspected to have cancer and did not have biopsy).

Independent variables	Patients who had biopsy in the clinic (n, %)	Patients who did not have biopsy in the clinic (n, %)	P value
Age (mean)	59.27	46.59	<0.001
Female sex	325 (95.0%)	23 (95.8%)	0.95
Found lump to be hard and craggy on self examination	8 (36.4%)	51 (14.8%)	0.10
Dimpling and puckering of the skin	1 (4.7%)	13 (3.7%)	0.85
Average age when the patient had first child	24.81	25.57	0.85
Age < 50 at menopause (% of total women who had their menopause	9 (40.9%)	74 (24.3%)	0.85
Average time (months) for breastfeeding	7.83	8.34	0.83
Family history of first degree relative with breast cancer	3 (12.5%)	30 (8.7%)	0.43
Family history of first degree relative with ovarian cancer	1 (4.2%)	11 (3.2%)	0.77
Past history of breast cancer	2 (8.3%	18 (5.3%)	0.52
Previous breast implants	1 (4.2%)	16 (4.7%)	0.90
Average time (years) for usage of hormone contraception/replacement therapy	7.05	4.67	0.06
History of diabetes	3 (12.5%)	21 (6.1%)	0.22
History of obesity	6 (25.0%)	62 (18.1%)	0.49

Software based on the same clinical guidelines agreed almost completely with surgeons who independently reviewed the patient data and applied the same guidelines: the sensitivity and specificity of the online software of identifying patients who required urgent clinic review was 100% and 98% respectively.

## 4 Discussion

The data suggests that a significant amount of referrals by GPs to urgent services were inappropriate according to NICE Guidelines. The most common symptom for inappropriate referral not needed to be seen in secondary care was breast pain (64.9%), which also was the second most common symptom for overall referrals. The online application consists of six questions related to ‘red flag breast symptoms’ and has been validated by comparison with outcomes given by breast surgeons based on NICE Guidelines.

One of the reasons to conduct the questionnaire was to identify any socio-demographic factors related to the patients who had biopsy in the clinic, which means that these certainly required urgent clinic review. This was different from evaluating factors related to breast cancer which has been thoroughly reviewed in the literature before. The biopsied lesions in the clinic will also include indeterminate lumps and symptoms with diagnostic uncertainty. From the response of the questionnaire, age was the only significant factor associated with having a biopsy in the clinic and that was included in the online application algorithm.

With self-referral pathways becoming commonplace in healthcare, there is already data testing its efficacy, and overall it appears to give high levels of both patient and clinician satisfaction [5]. There is data to support that online based self-referral applications are user friendly in order to be applicable to the wide demographic, which is necessary in health care [8]. Perhaps due to increasing campaigns with regards to detection of potential cancer symptoms, patients are now considered more educated and empowered with their own health [9].

The application has positive implications in both primary and secondary care. It will reduce appointment loads for GPs by allowing direct referrals to the specialty. It will also reduce delay from time of first noticing symptoms to specialist assessment/imaging as due to increased workload in primary care there is often significant wait for a GP appointment. In addition, most patients with breast symptoms experience anxiety the time they wait to be seen and re-assured by the breast surgeon [[1], [2], [3],^10^]. The application will aid patients in seeking help quickly when needed as well as potentially reducing the number of intimate examinations which itself is often a source of delay in seeking medical attention [10,11].

There is a potential for the online application to be cost effective. Based on the data that 39.6% of referrals did not warrant an appointment in secondary care, the application will reduce inappropriate attendance to urgent clinics while ensuring that 100% of patients with red flag symptoms are seen on an urgent basis. The application also provides a ‘safety net’ by suggesting patients whose symptoms can be managed in the community to visit their GPs. The system will allow patients to access the ‘choose and book’ system in order to book an appointment directly. This will have a positive financial impact as it will reduce the cost of missed appointments as well as the cost of an urgent referral to secondary care.

The next step in developing this application would be to implement this system in the community. It is also worth considering the potential to expand the concept to other common presenting complaints within surgical specialities. This will allow GP's to focus their time on the management of more common medical and chronic conditions and therefore provide better continuity of care for their patients. The role of artificial intelligence in healthcare is a broad field with a huge potential for future research [12]. Much research is already ongoing with regards to cancer detection in terms of interpretation of radiological images; this can be expanded into patient referral pathways.

The GPs have a low threshold to refer patients to the breast clinic [1]. The GP training has been shortened in recent times and their exposure and experience in surgical niche specialty, like breast onco-plastics, is limited. This makes decision making for them difficult and, hence, they have the tendency to refer patients to secondary care with benign breast conditions to rule out breast cancer. The concept of ‘gate-keeping’ role in primary care is not justified in this scenario and puts more strain on the system. Moreover, the waiting times for GP review are getting worse for scheduled appointments and more patients are pushed to book for urgent GP review or out-of-hours GP review. This is more burdensome on the urgent GP service and leads to lack of appropriate utilisation of the service. The majority of the GP assessments are based on the management of chronic conditions. The use of online application for self-referral for the patients with symptoms relating to sub-specialty of surgery will ease benefit the primary as much as it would to the secondary care.

There are certain limitations of the study which need to be taken into account when interpreting data. Collection of retrospective data does give potential for selection bias. There are currently no online or phone applications with the same concept to allow an accurate comparison for the application that we developed. However, we have validated our application using outcomes from surgeons. When considering population demographics, some patients may be reluctant or unfamiliar with using digital applications and this itself may cause a delay in presentation. However assistance from relatives or healthcare staff may aid to bypass this. The elderly patients not used to the technology should still be offered to go to the GP for breast symptoms, but GP can then use the same system in their clinic to refer the patient to the breast unit. The application is also only designed to triage patients at first presentation so is not currently able to be used by those with recurrent breast conditions or surgical follow-up. Furthermore there is a potential risk that the self-referral can increase the workload on the secondary care as it will bypass the ‘gate-keeping’ role of the primary care. However, previous studies have shown that GPs have a low threshold in referring these patients to secondary care. The self-referral system was introduced in other countries and it did not lead to increase in the number of referrals to the specialist care.[13] The use of technology for the elderly patients may be cumbersome but most of these patients are looked after by the family members, carers or visit by the primary healthcare professionals. The carers of these patients can make the referral online on their behalf if the patient or their carers suspect any breast problem.

## 5 Conclusion

A large proportion of patients referred on a ‘two week wait’ pathway were inappropriately referred. GP's have a low threshold to refer patients on an urgent basis which contributes to both an increased workload on these urgent secondary care services, as well as causing a high level of patient anxiety. Based on national guidelines we have developed an application to streamline patients with breast signs and symptoms through a self-referral pathway. This will allow patients to be categorized to be seen on either; an urgent basis, a routine basis or to be seen by their general practitioner.

## Funding

No funding was obtained for the study.

## Consent

Informed consent was obtained from patients.

## Ethical approval

Ethical approval for the study was obtained by the Department of Audit and research at Southend University Hospital.

## Author contribution

1) Mr Ahsan Rao conceptualized and designed the study, helped in data collection and managed the data analysis. He also contributed in writing of the article/manuscript.
2) Dr Humayun Razzaq did data collection and compilation, helped in designing the application, helped in analysis and contributed in writing of the article and referencing.
3) Dr Ben Panamarenko helped in data collection, formulation of algorithms, writing of manuscript and referencing.
4) Professor Alex Bottle helped with the study design, gave valuable input towards concept of the study, contributed towards data analysis as well as overall execution of the project.
5) Professor Azeem Majeed had valuable input in critical review of the study design, setting of parameters, formulation of algorithms and proof reading of manuscript.
6) Miss Emma Gray supervised the overall execution of the project with valuable inputs in study design, making sure algorithms are based on latest evidence, helped in analysis of data and proof reading of the final manuscript as well as its editing.

## Registration of Research Studies

1. Name of the registry: researchregistry
2. Unique Identifying number or registration ID: researchregistry6755
3. Hyperlink to your specific registration (must be publicly accessible and will be checked): https://www.researchregistry.com/browse-theregistry#home/registrationdetails/607b6c983e9570001be5ef9b/

## Guarantor

Mr Ahsan Rao, Department of Breast Oncoplastics, Addenbrookes Hospital Cambridge.

Dr Humayun Razzaq, Department of Colorectal and General surgery, Southend University Hospital

## Declaration of competing interest

Mr Ahsan Rao, who is the lead author of the study, is also one of the managing directors of the IT company Gnovatech pvt limited, which helped to design the software.

## Footnotes

Appendix A Supplementary data to this article can be found online at https://doi.org/10.1016/j.amsu.2021.102372.
