# Supplementary material for: Online application for self-referral of the patients with breast symptoms
Source: Ann Med Surg (Lond). 2021 May 8;66:102372. doi: 10.1016/j.amsu.2021.102372 (PMC8141459; doi:10.1016/j.amsu.2021.102372)
Supplement: Multimedia component 1 [file mmc1.docx]

***Appendix 1. Online questionnaire provided to the patients for data collection***

*Patient Name*

*Date Of Birth*

*Sex*

*Main Complaint*

*Where do you feel the lump(s)?*

1. *On one side 2. On both sides 3. No lumps felt*

*Does the size of lump(s) change with menstrual cycle ?*

1. *Yes 2. No*

*Is the swelling or lump on the breast red, hot and tender?*

1. *Yes 2. No*

*Is there any ulcer of the skin over the lump ?*

1. *Yes 2. No*

*Is the lump hard and rough textured ?*

1. *Yes 2. No*

*What is the nature of discharge ?*

1. *Clear 2. Pus 3. White 4. blood*

*What is the frequency of discharge ?*

1. *More than twice a week 2. Less than twice a week*

*Does the severity of the pain change with the menstrual cycle ?*

1. *Yes 2. No*

*Is there any dimpling or puckering of the skin ?*

1. *Yes 2. No*

*Number of children ?*

*What was your age when you had your first child ?*

*Age at menopause?*

1. *Age < 50 2. Age > 50*

*How long (in months) you have breastfed for ?*

*Do you have any first degree relatives (parents, children, siblings) with breast cancer ?*

1. *Yes 2. No*

*Do you have a first degree relative (parents, children, siblings) with ovary cancer ?*

1. *Yes 2. No*

*Do you have a past medical history of breast cancer ?*

1. *Yes 2. No*

*Do you have a past medical history of non-cancer breast condition ?*

1. *Yes 2. No*

*Do you have a past medical history of ovary cancer ?*

1. *Yes 2. No*

*Have you noticed any lump in the armpit ?*

1. *Yes 2. No*

*Did you have previous breast surgery ?*

1. *Yes 2. No*

*For how many years in total have you taken hormonal contraception (pills, implant etc) or hormone replacement therapy (HRT)?*

*Do you have a history of diabetes ?*

1. *Yes 2. No*

*Do you take any medication or steroid pills that can suppress your immune system ?*

1. *Yes 2. No*

*How many pregnancies did you have that did not continue to live ?*

*How long have (in years) you been diagnosed with any mental health condition? if not, then enter 0 ?*

*Did you have a needle biopsy in the clinic ?*

1. *Yes 2. No*

*Have you been diagnosed with obesity or consider yourself obese (BMI > 30) ?*

1. *Yes 2. No*

***Appendix 2. Algorithm used to create software outcome based on National and local hospital trust guidelines***

*Patients requiring urgent 2 week wait review by the breast surgeons*

*Female, aged > 30 years + breast lump or lump in the axilla*

*Female, aged > 50 years + Nipple discharge/retraction/ulceration/distortion/rash*

*Male, aged > 50 years, unilateral breast lump*

*Any dimpling, puckering or ulcer of skin*

*Any past medical history of breast cancer*

*Patients requiring routine clinic review by the breast surgeons*

*Female, aged < 30, breast lump or axillary lump that is tender or rapidly growing in size*

*Female, aged < 50 years, Nipple retraction/ulceration/distortion/rash*

*Female, aged < 50 years, Nipple discharge which is bloody or stain clothes or happens more than twice a week*

*Male, aged < 50, unilateral breast lump*

*Breast pain for more than 2 months and not settling on conservative management*

*Skin rash on breast or nipple.*

*Patients to be seen by the GP*

*Female, aged < 30, breast lump/axillary lump*

*Female, aged < 50, Nipple discharge*

*Male, bilateral breast lump*

*Breast pain < 2 months duration*

*Large breast, or small breast, or injury to breast*

*General concerns about breast cancer*

*Patients to be seen in the family history clinic by specialist nurse*

*Family history of breast cancer*
